# Quality of life, health-related quality of life, and associated factors in Huntington’s disease: a systematic review

**DOI:** 10.1007/s00415-022-11551-8

**Published:** 2023-01-30

**Authors:** Pearl J. C. van Lonkhuizen, Wiebke Frank, Anne-Wil Heemskerk, Erik van Duijn, Susanne T. de Bot, Alzbeta Mühlbäck, G. Bernhard Landwehrmeyer, Niels H. Chavannes, Eline Meijer, the HEALTHE-RND consortium, Niels H. Chavannes, Susanne T. de Bot, Pearl J. C van Lonkhuizen, G. Bernhard Landwehrmeyer, Franziska Steck, Jiří Klempíř, Romama Konvalinková, Eva Bezuchová, Kristýna Dolečková, Olga Klempířová, Jan Roth, Olga Ulmanová, Ferdinando Squitieri, Sabrina Maffi, Eugenia Scaricamazza, Simone Migliore, Chiara Di Giorgio, Barbara D’Alessio, Melissa Casella, Jennifer Hoblyn, Muthukumaran Thangaramanujam, Tom Burke, Emer O’Malley, Stephen McKenna, Ian McKenna, Jeanette Thorpe, Anna Coffey, Ramona Moldovan, Peter Foley, Jacqueline Kerr

**Affiliations:** 1 Department of Public Health and Primary Care, Leiden University Medical Center, PO Box 9600, 2300 RC Leiden, The Netherlands; 2 National eHealth Living Lab, Leiden University Medical Center, Leiden, The Netherlands; 3 Huntington Center Topaz Overduin, 2225 SX Katwijk aan Zee, The Netherlands; 4 Department of Neurology, University Hospital Ulm, 89081 Ulm, Germany; 5 Department of Psychiatry, Leiden University Medical Center, 2300 RC Leiden, The Netherlands; 6 Department of Neurology, Leiden University Medical Center, 2300 RC Leiden, The Netherlands; 7 Department of Neuropsychiatry, kbo-Isar-Amper-Klinikum, 84416 Taufkirchen (Vils), Germany; 8 Department of Neurology and Center of Clinical Neuroscience, 1st Faculty of Medicine, Charles University and General University Hospital in Prague, 12821 Prague, Czech Republic

**Keywords:** Huntington’s disease, Quality of life, Health-related quality of life, Patient-reported outcome measures, Systematic review

## Abstract

**Background:**

Huntington’s disease (HD) is a genetic, neurodegenerative disease. Due to the progressive nature of HD and the absence of a cure, (health-related) quality of life ((HR)QoL) is an important topic. Several studies have investigated (HR)QoL in HD, yet a clear synthesis of the existing literature is lacking to date. We performed a systematic review on self-reported (HR)QoL, and factors and intervention effects associated with (HR)QoL in premanifest and manifest HD gene expansion carriers (pHDGECs and mHDGECs, respectively).

**Methods:**

PubMed, EMBASE, Web of Science, and PsycINFO were searched systematically from September 17th, 2021, up to August 11th, 2022. Methodological and conceptual quality of the included studies was assessed with two appraisal tools.

**Results:**

30 out of 70 eligible articles were included. mHDGECs experienced lower (HR)QoL compared to pHDGECs and controls, whereas mixed findings were reported when compared to other neurological diseases. Several factors were associated with (HR)QoL that might contribute to lower (HR)QoL in mHDGECs, including depressive symptoms, physical and psychological symptoms, lower functional capacity, lower support, and unmet needs. Multidisciplinary rehabilitation programs and a respiratory muscle training were beneficial for (HR)QoL in mHDGECs.

**Discussion:**

(HR)QoL is experienced differently across the course of the disease. Although (HR)QoL is key for understanding the impact of HD and the effect of symptomatic treatment, there is a need to improve the methodological and conceptual shortcomings that were found in most studies, especially regarding the conceptual clarity when reporting on QoL and HRQoL. Suggestions for strengthening these shortcomings are provided in this review.

**Supplementary Information:**

The online version contains supplementary material available at 10.1007/s00415-022-11551-8.

## Introduction

Huntington’s disease (HD) is a rare, autosomal dominant neurodegenerative disease, affecting an estimated 10–14 individuals per 100,000 within the Western population [1–3]. HD is caused by a cytosine–adenine–guanine (CAG) repeat expansion in the *Huntingtin* (*HTT*) gene [2, 3]. Individuals with > 39 CAG repeats will develop HD at some point during their lives. Offspring of an HD affected parent has a 50% chance of inheriting the gene expansion [3]. The clinical picture of HD is characterized by progressive motor, cognitive and neuropsychiatric symptoms. The disease course consists of various stages, including the premanifest phase (i.e., pre-symptomatic and prodromal phase) [2, 4, 5] in which modest symptoms can appear up to 10–15 years prior to the onset of characteristic motor symptoms in the manifest phase [2–6]. After motor symptom onset, which usually occurs between 30 and 50 years of age [1, 6], life expectancy ranges between 15 and 20 years [2]. Due to the absence of a cure, the gradual progression of the disease and the increasing need for care, treatment is mainly focused on the management of symptoms.

To date, the physical consequences of HD have been well described [1, 3, 6], shifting the focus towards assessment of more patient-centered outcomes including the impact of the disease on quality of life (QoL) [2, 3, 7, 8]. The QoL literature consists of different concepts related to QoL, including health status and health-related QoL (HRQoL), yet their operational definitions remain debatable [9, 10] and further clarification on the concepts used is desired when reporting QoL-related findings.

Health status refers to an individual’s functioning on physical, mental and social aspects of life [11, 12] and can be reported by the individual as well as by others. QoL goes beyond a mere description and includes a subjective perception and evaluation of an individual’s functioning on these domains [10]. The World Health Organization (WHO) defines QoL as follows: “individuals’ perception of their position in life in the context of the culture and value systems in which they live and in relation to their goals, expectations, standards and concerns” [11] (p. 1405). The concept of QoL, therefore, extends to more dimensions, including the spiritual, material, financial, environmental, and cultural dimensions [11], and focuses on a more holistic perspective compared to health status and HRQoL. HRQoL is not well defined in the literature and often shows considerable overlap with the definitions of QoL and/or health status [9]. HRQoL can be considered a more restrictive definition of QoL in which only health-related factors are evaluated and other non-health-related factors are excluded (e.g., culture, housing, and finances) [10].

Despite the differences between these QoL-related concepts and the lack of clear definitions, there is a variety of instruments available that measure QoL-related concepts, including generic and disease-specific assessments ranging from single item measures to full scales. A commissioned review has been undertaken to enrich our understanding of different questionnaires used to assess HRQoL in HD gene expansion carriers (HDGECs) [13]. Although the authors of this review provided a comprehensive overview on the use and properties of 14 patient-centered questionnaires for HRQoL assessment in HD, they did not focus on the outcomes of the questionnaires, i.e., how HRQoL was actually evaluated by HDGECs themselves. The focus on self-perceived (HR)QoL is especially important in HD given the serious consequences of HD and the lifelong influence of the disease on the affected individual. Over the past years, several studies examined the (HR)QoL of HDGECs, as well as factors associated with (HR)QoL. However, there is a lack of literature that summarizes these findings to date. A synthesis of available research can help to better understand (HR)QoL over the course of the disease and can provide directions for treatment and future research.

The aim of this study was to systematically review the current knowledge on the self-reported perceptions of (HR)QoL in both premanifest and manifest HDGECs. In addition, we aimed to identify factors associated with and potential intervention effects for (HR)QoL in these groups.

## Methods

### Search strategy and procedure

The search strategy was built collectively with an information specialist of the library service of Leiden University Medical Center in the Netherlands. A systematic literature search was performed in PubMed, EMBASE, Web of Science, and PsycINFO from September 17th, 2021, up to August 11th, 2022. The search string included the key term “Huntington’s Disease” (including related terms “Huntington”, “Huntington’s”, and “Huntingtons”) in combination with the key term “Quality of Life” (including related terms “QoL”, “HRQoL”, and “Life Quality”). See Supplementary materials S1 for the full search string along with its adaptations to each database. Cross-references of relevant retrieved articles were checked to identify any potential additional articles.

Articles were independently screened on title and abstract by the first (PL) and second author (WF). Eligible articles, as well as articles of which eligibility was unclear, were obtained in full text and reviewed independently by the same authors (PL and WF). Any disagreements about the eligibility of full-text articles were resolved in consultation with the third (AH) and last author (EM) by consensus through discussion. Full-text analysis and data extraction was performed by the first author (PL) and checked and complemented, when necessary, by the second author (WF). The following data were extracted per study: authors, publication year, database of extraction, design and aim, country, measured construct, method of QoL assessment, HD subsample, number of participants per sample, age and sex of participants, inclusion criteria, other included samples, other outcome measures, main findings regarding (HR)QoL, and strengths and limitations.

**Fig. 1:**
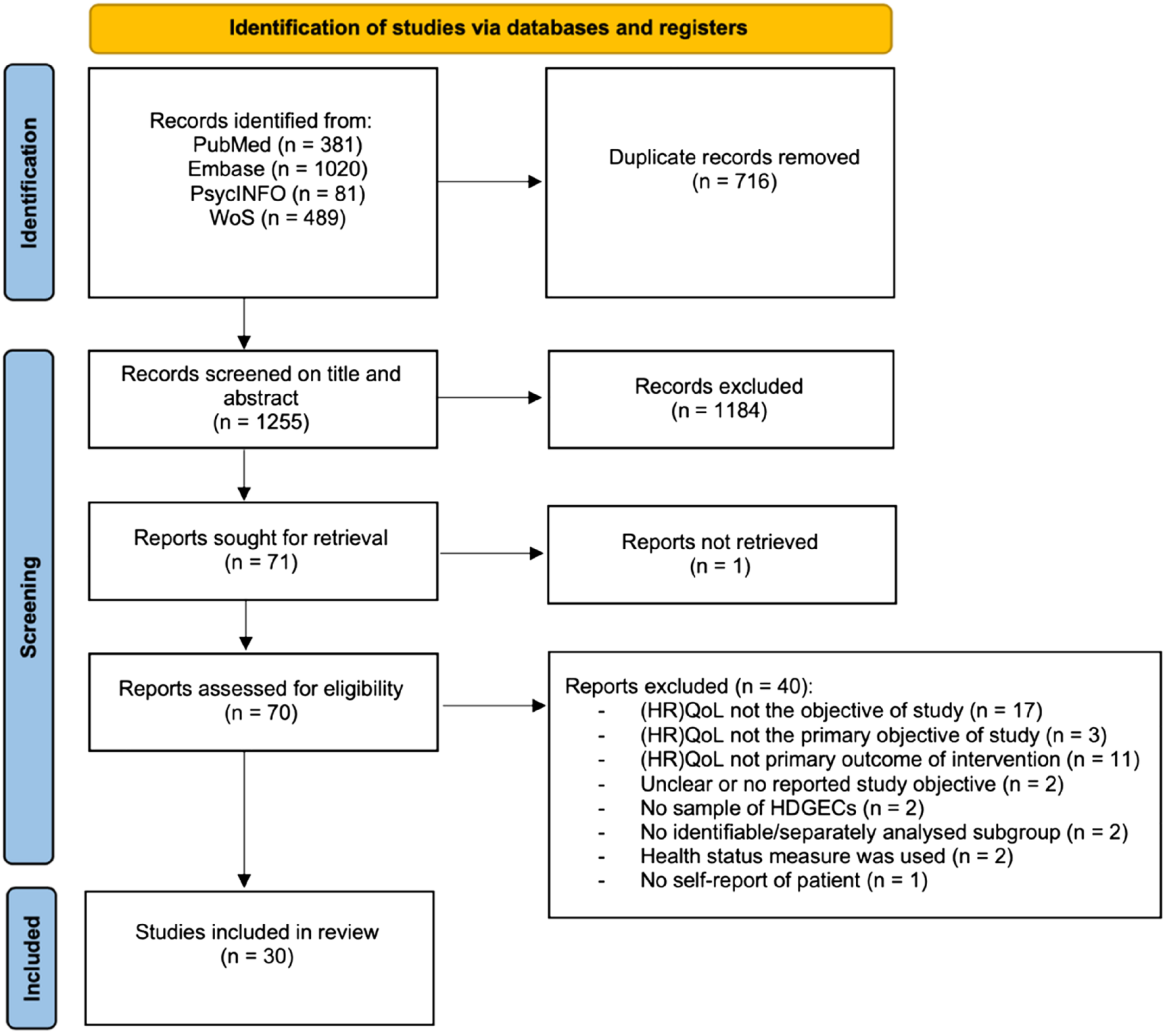
Flow diagram of article selection process

This review was conducted in accordance with the PRISMA guidelines to ensure transparency in performing and the reporting of the review [14]. A flow diagram of the article selection process, conforming to the PRISMA guidelines, is depicted in Fig. 1.

### Eligibility criteria

Articles that met the following criteria were included in this systematic review: (1) the primary objective was to describe or evaluate self-reported (HR)QoL and/or its associated factors in HDGECs; (2) the study sample exclusively involved or included an identifiable and separately analyzed subsample of genetically and/or clinically confirmed (i.e., premanifest and/or manifest) HDGECs; (3) (HR)QoL was measured quantitatively and/or qualitatively; (4) in case of intervention studies, (HR)QoL needed to be the primary or main outcome measure; (5) the article was peer-reviewed and written in English, Dutch or German. To provide a full synthesis of (HR)QoL research in HD, we included all articles that aimed to capture QoL and/or HRQoL regardless of definition or assessment method used. There was no restriction on publication dates.

Articles were excluded from this review if (1) they involved persons at risk of or tested negative for HD; (2) they assessed health status; (3) they focused on the development and/or validation of questionnaires; (4) they were case reports or not originally published empirical research articles; (5) the full text was unavailable.

### Methodological quality assessment

The methodological quality of the included studies was assessed using the mixed-methods appraisal tool (MMAT) (version 2018) designed for reviews that include qualitative, quantitative and mixed-methods studies [15]. We also evaluated the conceptual and methodological clarity of the included articles with a critical appraisal tool for reviewing the quality of QoL studies developed by Gill and Feinstein [16] and further refined by Moons et al. [17].

For the MMAT [15], each article was appraised according to the five criteria of the corresponding study category (i.e., qualitative, quantitative randomized-controlled trial, quantitative non-randomized, quantitative descriptive, and mixed-methods). Rating options included ‘yes’, ‘no’ or ‘can’t tell’, with the latter option applying to articles that did not report appropriate or clear information in order to answer ‘yes’ or ‘no’. For interpretative purposes and the computation of an overall score, ‘can’t tell’ was considered as a ‘no’. Item 3.5 ‘Intervention is administered (or exposure occurred) as intended’ was substituted with item 4.5 ‘Statistical analysis is appropriate to address the aim’. Item 4.5 was considered more appropriate for the quantitative non-randomized studies included in this review as the majority of these studies were non-intervention studies and the exposure status of participants (i.e., carrying the HD gene expansion) was fixed. Regarding the tool for reviewing QoL studies [16, 17], each article was appraised on ten criteria with the rating options: ‘yes’, ‘no’, or ‘not applicable’. As we include both QoL and HRQoL studies, we applied the criteria for QoL studies to the included HRQoL studies as well.

The included articles were independently appraised on the criteria lists of both tools by the first (PL) and second (WF) author. Any disagreements were resolved by consensus through discussion. An overall quality score was computed by dividing the sum of the items that an article met by the number of items for which the article was eligible to be appraised. The score was then multiplied by 100, resulting in an overall quality score ranging from 0% (none of the criteria met) to 100% (all of the criteria met) (see Table 1).

**Table 1:** Characteristics of included studies

First author (year)	Country	Study design	HD sample, *N*	Age (mean)	Gender (male, %)	Other groups, *N*	Statistical comparison with(in) group(s)	Study aim	Intended concept	Method of (HR)QoL assessment	Appraisal score (MMAT, QoL)
(HR)QoL evaluation studies											
Chapman (2002) [33]	UK^a^	Qualitative—phenomenological approach	NS^b^, 21	42	43%	CF, 31 Unaffected carriers/family, 11	No	To present perceptions of health and QoL in individuals with early- or late-onset genetic conditions	QoL +	Individual interviews	60%, 0%
Ready (2011) [32]	USA	Qualitative—consensual qualitative research approach	pHDGECs, 9	38	44%	Companions, 6	No	To explore perceptions of QoL in pHDGECs and companions	QoL +	Individual interviews	80%, 0%
Calvert (2013) [29]	UK	Quantitative non-randomized—analytical cross-sectional design	mHDGECs^b^, 53	57	45%	rLTND, 213	Yes	To assess HRQoL and access to supportive health and social care in individuals with rare long-term neurological conditions	HRQOL +	EQ-5D^c^	40%, 13%
Carlozzi (2013) [36]	USA	Qualitative—theoretical framework/participatory action approach	mHDGECs^d^, 24	49	NR	At-risk/prodromal, 16 Caregivers, 17 HD clinicians, 25	No	To explore domains that reflect HRQOL in HD and to identify pre-existing measures of HRQoL relevant to HD	HRQoL +	Focus groups	100%, 100%
Chisholm (2013) [40]	USA	Quantitative non-randomized—analytical cross-sectional design	pHDGECs, 37 mHDGECs, 31	44 47	27% 56%	At-risk, 65 Healthy controls, 95	Yes	To evaluate and compare well-being (including QoL) in persons at-risk for HD, pHDGECs, mHDGECs, and controls	QoL +	Single item (item not defined)	40%, 13%
Read (2013) [26]^e^	Multinational (not defined)	Quantitative non-randomized—analytical cross-sectional design	pHDGECs, 118 group A, B^f^ mHDGECs, 117 group C, D^g^	41, 40 47, 51	46%, 44% 39%, 55%	Partners, 84 Siblings, 36	Yes	To explore HRQoL in pHDGECs and mHDGECs compared to a control group of individuals from the HD community	HRQoL + −	SF-36^c^, QoLI^c^	80%, 25%
Dorey (2016) [35]	Spain	Quantitative descriptive—descriptive cross-sectional design	mHDGECs, 55	50	49%	Caregivers, 55	No	To explore and compare HRQoL in mHDGECs using a disease-specific and generic questionnaire	HRQoL +	EQ-5D^c^ H-QoL-I^h^	40%, 13%
Varda (2016) [23]^e^	Cyprus	Quantitative non-randomized—analytical cross-sectional design	pHDGECs, 9 mHDGECs, 23	53^i^	38%^j^		Yes	To evaluate QoL in individuals with HD using a standardized HRQoL questionnaire	QoL −	EQ-5D^c^	100%, 38%
Sherman (2019) [37]	USA	Qualitative—theoretical framework/participatory action approach	mHDGECs^d^, 24 Early (*n* = 8) Advanced (*n* = 16)	50 50	37% 56%	At-risk/prodromal, 16 Caregivers, 17 HD clinicians, 25	No	To explore experiences of chorea and its impact on everyday functioning and HRQoL in HD stakeholders	HRQoL +	Focus groups	60%, 0%
Exuzides (2022) [42]	USA	Quantitative non-randomized—analytical cross-sectional design	mHDGECs, 41	46	32%	HD carers, 80 PD, 118 PD carers, 385 Controls, 123 Control carers, 240	Yes	To evaluate the unique and shared burdens, including the impact on HRQoL, of mHDGECs and HD care partners, compared to Parkinson’s Disease and the general population	HRQoL +	EQ-5D^c^	100%, 25%
Engels (2022) [38]	NL	Qualitative—descriptive and explorative approach	mHDGECs, 36	NR^k^	39%	Family members, 11 Nurses, 36	No	To explore and describe perceived QoL of HD patients from the perspective of mHDGECs, family members, and nursing staff	QoL +	Individual interviews^l^	100%, 50%
Studies examining associations of (HR)QoL											
Ready (2008) [39]^e^	USA	Quantitative non-randomized—longitudinal design	mHDGECs, 22	47	73%	Caregivers, 22	No	To examine relationships between patient QoL, caregiver QoL, and symptoms of HD	QoL +	Single item (Overall, how would you rate your QoL?)	40%, 50%
Ho (2009) [45]	UK	Quantitative non-randomized—analytical cross-sectional design	mHDGECs^b^, 70	50	50%			To examine which variables are linked with HRQOL in mHDGECs	HRQoL +	SF-36^c^	60%, 25%
McCabe (2009a) [34]	Australia^a^	Quantitative non-randomized—longitudinal design	NS^b^, 26	59	69%	PD, 100 MND, 52 MS, 79	Yes	To identify predictors of economic pressure and QoL in individuals with progressive neurological illness	QoL +	WHOQoL-BREF^c^	40%, 25%
Banaszkie- wicz (2012) [24]	Poland	Quantitative non-randomized—analytical cross-sectional design	mHDGECs^b^, 80	48	NR	Caregivers, 80	No	To determine predictors of patients’ disability, QoL and caregivers' burden in HD	QoL −	SF-36^c^	60%, 13%
Eddy (2013) [19]	UK	Quantitative non-randomized—analytical cross-sectional design	mHDGECs^b^, 20	54	60%			To examine associations between QoL, behavioral and psychiatric symptoms, and executive function in mHDGECs	QoL −	SF-36^c^	20%, 25%
Eddy (2014) [20]	UK^a^	Quantitative non-randomized—analytical cross-sectional design	mHDGECs, 30	55	60%	Healthy controls, 20	No	To assess associations between spatial/social perspective taking and executive deficits, everyday perspective taking, motor symptoms, functional capacity and QoL in mHDGECs	QoL −	SF-36^c^	40%, 13%
Brugger (2015) [43]	Austria	Quantitative non-randomized—analytical cross-sectional design	mHDGECs^b^, 80	NR	54%			To assess the impact of disease characteristics on HRQoL in HD	HRQoL +	SF-36^c^	60%, 25%
van Walsem (2016) [30]	Norway	Quantitative non-randomized—analytical cross-sectional design	mHDGECs, 84	57	56%			To assess ATC use across disease stages in mHDGECs and to examine the associations between ATC and HRQoL	HRQoL +	EQ-5D^c^	80%, 13%
Fritz (2018) [44]	USA	Quantitative non-randomized—analytical cross-sectional design	pHDGECs, 193 mHDGECs, 278	49 ^j^	40% ^j^			To examine associations between apathy, functional status, physical function, cognitive function, behavioral status/emotional function and HRQoL in HD	HRQoL +	EQ-5D^c^ RAND-12^c^ WHODAS^c^	60%, 13%
Zielonka (2018) [22]^e^	Mixed (EU)	Quantitative non-randomized—analytical cross-sectional design	mHDGECs, 1166	NR for subsample	52%			To compare if gender differences in motor, cognitive and behavioral symptoms affect function and how functional impairment affects QoL in mHDGECs	QoL −	SF-36^c^	100%, 13%
Ready (2019) [25]	USA^a^	Quantitative non-randomized—longitudinal design	pHDGECs, 50 mHDGECs, 272	52 ^j^	46% ^j^			To assess the relationship between positive affect and HRQOL outcomes in HD and whether these associations were moderated by functional status	HRQoL + −	PROMIS^c^ Neuro-QoL^c^ HDQLIFE^h^	80%, 13%
Studies investigating both (HR)QoL and its associated factors											
Licklederer (2008) [21]	Germany	Quantitative non-randomized—analytical cross-sectional design	pHDGECs, 54 mHDGECs, 15	NR for subsamples	NR for subsamples	Non-carriers, 52	Yes	To examine mental health and QoL in HD, to compare mental health between non-carriers, pHDGECs and mHDGECs, and to determine predictors of mental health and QoL	QoL–	SF-12^c^	80%, 25%
McCabe (2009b) [41]	Australia	Quantitative non-randomized—analytical cross-sectional design	mHDGECs^b^, 48	57	63%	PD, 143 MND, 120 MS, 112	Yes	To examine differences in mood, symptoms, and QoL in people with progressive neurological illness, and to examine predictors of mood and QoL	QoL +	WHOQoL–BREF^c^	60%, 13%
van Walsem (2017) [31]	Norway	Quantitative non-randomized—analytical cross-sectional design	mHDGECs, 84	57	56%			To describe HRQoL in mHDGECs and to examine the relationship of unmet needs for healthcare and social support services with HRQoL	HRQoL +	EQ-5D^c^	80%, 38%
Intervention studies											
A'Campo (2012) [18]	NL	Quantitative non-randomized—single-group pre–post-design	pHDGECs, 19 mHDGECs, 40	41 53	32% 65%	Partners pHDGEC, 14 Carers mHDGEC, 28	No	To evaluate the feasibility of the Patient Education Program for HD (of which the goal is to improve QoL)	QoL–	SF-36^c^	60%, 25%
Piira (2013) [27]	Norway	Quantitative non-randomized—single-group pre–post-design	mHDGECs, 37	52	49%			To evaluate the effects of a 1-year intensive, multidisciplinary rehabilitation program for mHDGECs (with HRQoL among the main outcome measures)	(HR)QoL^m^	SF-12^c^	60%, 13%
Piira (2014) [28]	Norway	Quantitative non-randomized—single-group pre–post-design	mHDGECs, 10	50	50%			To evaluate the effects of a 2-year intensive, multidisciplinary rehabilitation program for mHDGECs (with HRQoL among the main outcome measures)	(HR)QoL^m^	SF-36^c^	40%, 13%
Reyes (2015) [46]	Australia	Quantitative randomized-controlled trial—pilot study	mHDGECs, 18 training (*n* = 9) control (*n* = 9)	56 50	67% 56%			To evaluate the effects of a 4-month respiratory muscle training on pulmonary and swallowing function in mHDGECs (with QoL among the main outcome measures)	Swallow QoL +	Swal-QoL^c^	80%, 13%
Ringqvist (2021) [47]	Sweden	Quantitative non-randomized—cohort study	mHDGECs, 20	52	20%			To assess the effect and tolerability of a 25-day multimodal rehabilitation program for mHDGECs on psychiatric symptoms, HRQoL, and psychological health factors	HRQoL +	EQ-5D^c^	100%, 13%

Mean and percentages in table are rounded

*HD* Huntington’s disease, *N* sample size (refers to participants with complete (HR)QoL data. In case of intervention studies, the *N* refers to the number of participants with complete (HR)QoL data at baseline), *(HR)QoL* (health-related) quality of life, *MMAT* mixed-methods appraisal tool, *UK* United Kingdom, *NS* not specified, *CF* cystic fibrosis, *rLTND* rare long-term neurological conditions, *USA* United States of America, *pHDGECs* premanifest Huntington’s disease gene expansion carriers, *mHDGECs* manifest Huntington’s disease gene expansion carriers, *EQ-5D* EuroQoL 5D, *NR* not reported, *SF-36* Medical Outcomes Study 36-Item Short Form Health Survey, *H-QoL-I* Huntington Quality of Life Instrument, *NL* the Netherlands, *WHOQoL-BREF* World Health Organization Quality of Life Questionnaire short version, *PD* Parkinson’s disease, *MND* motor neuron disease, *MS* multiple sclerosis, *ATC* Assistive Technology for Cognition, *WHODAS* WHO Disability Assessment Schedule, Patient-Reported Outcomes Measurement Information System, *EU* European, *HDQLIFE* Huntington Disease Quality of Life, *SF-12* Medical Outcomes Study 12-Item Short Form Health Survey, *QoLI* Quality of Life Inventory, *RCT* randomized-controlled trial

^a^In case country of study/recruitment is not reported, country is based on author(s) affiliations

^b^Study sample not clearly reported; decisions were based on reported characteristics of the study sample. In case of reported disease onset/duration, we grouped participants as mHDGECs. NS refers to articles that did not include sufficient information on the study sample (i.e., “HD gene-positive individuals” for Chapman 2002 and “HD patients” for McCabe 2009a)

^c^Generic (HR)QoL instrument

^d^The study also included pHDGECs but not as a sub-identifiable group as pHDGECs were grouped together with persons at risk

^e^Study also reports relevant findings (e.g., evaluation of (HR)QoL or associated factors thereof) although this was not reported as part of the initial aim

^f^Group A: pHDGECs furthest from predicted age of HD onset (*n* = 61); Group B: pHDGECs closest to predicted age of HD onset (*n* = 57)

^g^Group C: mHDGECs in early stage I of the disease (*n* = 75); Group D: mHDGECs in early stage II of the disease (*n* = 42)

^h^Disease-specific (HR)QoL instrument

^i^Median age for combined sample of pHDGECs and mHDGECs

^j^Value reported for combined sample of pHDGECs and mHDGECs

^k^Age range (30–84 years) was reported

^l^The authors used others methods of data collection (i.e., interviews with family members and nurses, and qualitative observations of interaction between patients and nurses together), however only the results of the qualitative interviews with patients are reported as the data collection methods are not in accordance with our inclusion criteria)

^m^QOL and HRQOL was not used among the aim, but was used interchangeably in the main outcome measure section of the abstract and methods

^+^Intended construct of (HR)QoL corresponds with actual labeling of the tool

^−^Intended construct of (HR)QoL does not correspond with actual labeling of the tool

^+ −^Intended construct of (HR)QoL corresponds to some of the labels of the included tools

## Results

**Fig. 2:**
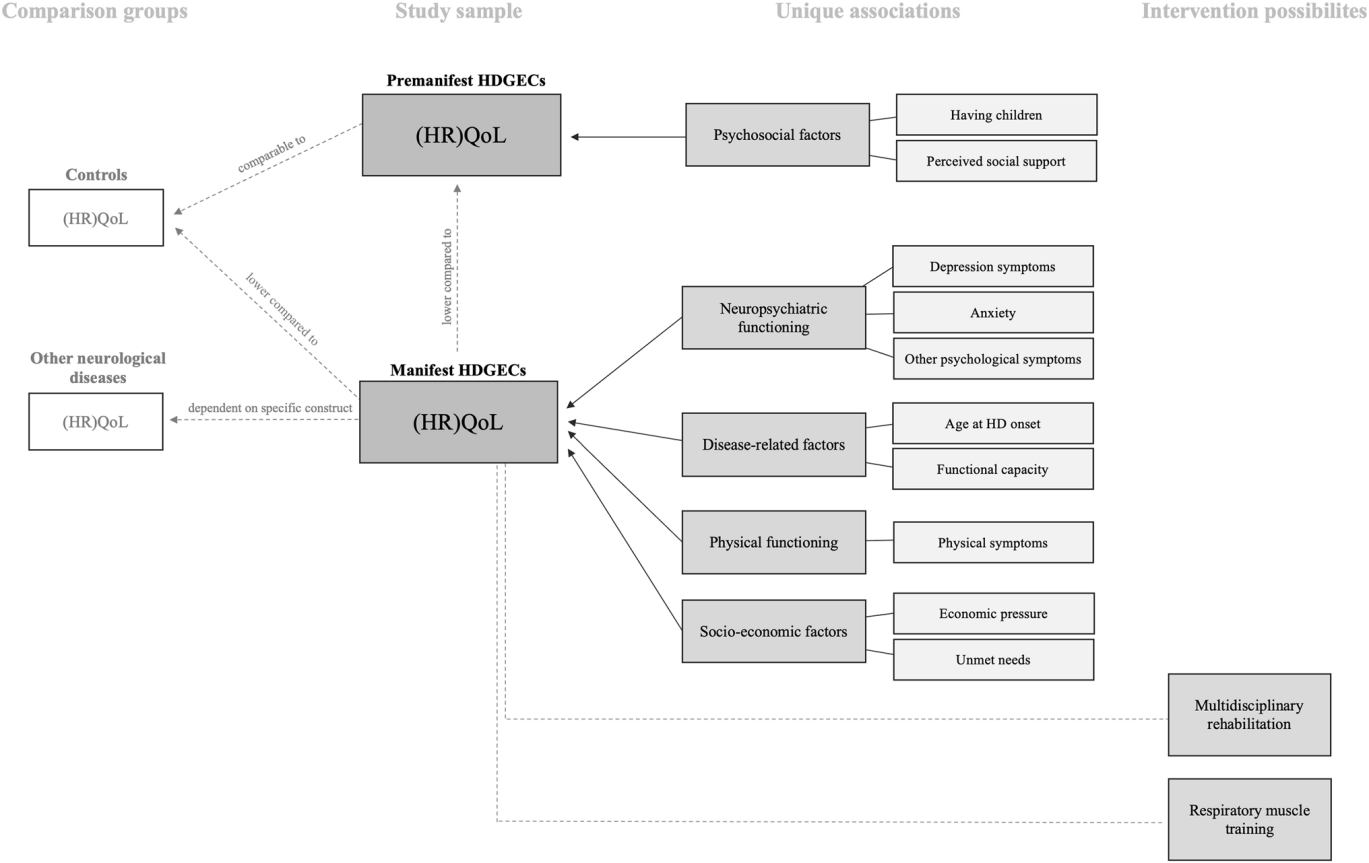
Main findings on (HR)QoL and its uniquely associated factors and intervention effects in HDGECs. Uniquely associated factors were only included in this figure if the majority of the studies examining that factor found a significant effect

The literature search identified 1255 unique records, of which 30 studies met the inclusion criteria for this review (see Fig. 1). The main findings of this review are displayed in Fig. 2.

### Methodological quality of included studies

The methodological quality of the included studies, as appraised with the MMAT [15], ranged from 20% (*n* = 1) to 100% (*n* = 6) (see Table 1). Most of the included studies involved quantitative research (*n* = 25), of which the majority (*n* = 15) scored ≤ 60%. The most frequent shortcomings across these studies were unclear information about the representativeness of the study sample (*n* = 13) and not accounting for confounders in the design and analysis (*n* = 13). The other remaining articles (*n* = 5) were defined by a qualitative design, with quality scores of 60% (*n* = 2) and ≥ 80% (*n* = 3). Limitations across these studies consisted of a lack of coherence between data sources, collection, analysis, and interpretation (*n* = 3) and insufficient interpretation of results based on the data (*n* = 2). No mixed-methods studies were performed.

Appraisal scores for the conceptual and methodological clarity [16, 17] of the included articles ranged from 0% (*n* = 3) to 100% (*n* = 1) (see Table 1). The majority of studies (*n* = 25) scored ≤ 25%, whereas only three studies scored ≥ 50%. When looking at the specific items, authors conceptually defined (HR)QoL in only 6 out of 30 articles and an explicit differentiation between QoL and HRQoL was given in only 2 out of 30 articles. 20 out of 25 studies with a quantitative design included a composite score for (HR)QoL and in 8 out of 25 studies participants rated their overall (HR)QoL on a single item measure. However, in only three studies the authors stated the (HR)QoL domains they considered important to measure, whereas four studies gave valid reasons for selecting the (HR)QoL instrument(s) of choice. In none of the included studies, participants could supplement items or indicate the personal importance of items. For the qualitative studies, these criteria were not applicable. All papers irrespective of methodological quality as appraised with both tools were included in this review. See Tables S2 and S3 in the Supplementary materials for a detailed overview of the compliance per item per study for both tools.

Although not covered in these appraisal tools, yet equally important, is the (lack of) correspondence between the intended construct and assessment method used. Of the 25 studies that quantitatively investigated (HR)QoL, 7 studies aimed to measure QoL but actually used a HRQoL questionnaire [18–24], whereas 2 studies aimed at measuring HRQoL and included a QoL questionnaire in addition to HRQoL questionnaires [25, 26]. Two studies interchangeably used the terms QoL/HRQoL throughout the abstract and introduction [27, 28]. For interpretative purposes, findings were categorized as reporting either on QoL and/or HRQoL according to the initial labeling of the used instrument. In case of qualitative studies, findings were grouped according to the intended construct in the study’s initial aim as these studies focused on (HR)QoL perceptions/experiences rather than quantifying a specific construct.

Moreover, studies were categorized according to their initial aim into: (HR)QoL evaluation studies, studies investigating associated factors of (HR)QoL, and/or intervention studies (see Table 1). Some studies reported additional findings that did not correspond with the study’s initial aim; these findings have been included when relevant. In addition, as some studies lacked a clear description of the included HD sample, HDGECs were categorized as either premanifest (i.e., pre-symptomatic and prodromal) or manifest (i.e., onset of clinical motor symptoms) based on the available reported information (such as disease onset/duration in case of mHDGECs). In some studies, a small minority of participants were not able to self-report their HRQoL and, therefore, assistance of an informant [29] or a proxy report was needed [24, 30, 31]. In two of these studies, removing these participants from the analysis did not change the findings [30, 31], whereas in the other two studies, it was unclear how the authors dealt with this [24, 29].

### Characteristics of included studies

All study characteristics are presented in Table 1. We included 11 (HR)QoL evaluation studies, 11 studies investigating associated factors of (HR)QoL, 3 studies that combined both evaluation and associated factors of (HR)QoL, and 5 intervention studies.

The majority of the studies were conducted in Europe (*n* = 13), followed by the USA (*n* = 8), the UK (*n* = 5), Australia (*n* = 3), and one study was carried out multinationally (not further specified). Recruited sample sizes of all included studies ranged from 9 to 1166 participants. Moreover, the majority of the studies included a sample of manifest HDGECs (mHDGECs) (*n* = 20), followed by studies among both premanifest (pHDGECs) and mHDGECs (*n* = 7). Only one study included a sample of solely pHDGECs [32] and for two studies the included sample was not clearly described or easily extractable from the text (i.e., ‘HD-gene positive individuals’ [33] and ‘HD patients’ [34]). Mean age of participants included across studies ranged from 38 to 59 years.

With regard to the 25 quantitative studies, (HR)QoL was assessed using either a generic and/or a disease-specific questionnaire (*n* = 23), or a single item rating of QoL (*n* = 2). In total, 11 different generic (HR)QoL questionnaires were used across the studies, of which the SF-36 was most commonly used (*n* = 9), followed by the EQ-5D [Visual Analogue Scale (VAS)] (*n* = 8). Only two studies included a disease-specific questionnaire (i.e., H-QoL-I, HDQLIFE) [25, 35]. In the five qualitative studies, (HR)QoL was explored via individual (un)structured interviews (*n* = 3) or focus groups (*n* = 2). Interviews were audio-recorded and transcribed in the majority of these studies [32, 33, 36, 37] and data were analyzed using thematic qualitative analysis [32, 33, 38], an expansion of both grounded theory and the multidimensional HRQoL theoretical framework developed by the WHO [36], or a combination of both thematic content analysis, grounded theory and matrix analysis [37].

### Quality of life

#### In HDGECs

Four studies assessed QoL in HDGECs only, without a control group. Three of these studies assessed QoL qualitatively [32, 33, 38], whereas the fourth study included a quantitative assessment over time [39].

All three qualitative studies differed in the level of detail in their reporting. In one study [33], HD-gene-positive individuals (not further defined, *n* = 21) indicated that their QoL was good, yet no other detailed findings for the HD subsample were reported [33]. In another study [32], frequently discussed QoL themes among a small group (*n* = 9) of pHDGECs were interpersonal relationships (31%) and coping with the HD gene mutation (27%). Other discussed themes included (witnessing) HD manifestations in others (10%), employment (6%), and spirituality (2%). pHDGECs talked both positively and negatively about interpersonal relationships, employment and coping with HD. The topic of spirituality was mostly discussed in positive terms, as opposed to the mainly negative statements used when discussing HD in others. Moreover, participants related their QoL to the present and talked about the present more positively than negatively. The past and the future were discussed in more negative terms, including statements referring to decisions about genetic testing, the initial response to a positive test result, and concerns about maintaining future employment [32]. The third study included a more detailed description of perceived QoL among mHDGECs admitted to an in-patient unit for advanced HD (*n* = 36) [38]. mHDGECs acknowledged that aspects of their identity might change with the progression of disease (e.g., roles in the family or at work) and that living with HD and its symptoms has implicated their well-being. Moreover, difficulty was experienced in dealing with loss of control, increased dependence, altered behavior, progression of the disease, and uncertainty [38]. mHDGECs emphasized the importance of maintaining their personal values and the unconditional acceptance of nurses regardless of changes in their behavior due to the progression of the disease. Preservation of identity and autonomy, meaningful and respectful relationships with nurses and their support in self-management in daily life were considered important for perceived QoL of mHDGECs, as were certain qualifications of nurses such as understanding, flexibility and patience [38].

One longitudinal study that quantitatively investigated QoL in 22 mHDGECs over time found no significant decrease in QoL between baseline and 6-month follow-up [39]. At follow-up, however, mHDGECs perceived their QoL at baseline as being worse than was actually the case at that time, suggesting a response shift [39].

#### Statistical comparison between HDGEC subgroups

One study compared QoL between different subgroups of HDGECs and showed that mHDGECs (*n* = 31) reported significantly lower QoL than pHDGECs (*n* = 37) [40].

#### Statistical comparison with other neurological diseases and controls

In total, three studies compared the QoL of HDGECs with that of other patient groups or controls [26, 40, 41]. One study found significant differences in QoL between different groups of neurological diseases [i.e., Parkinson’s disease (PD) (*n* = 143), motor neuron disease (*n* = 120), multiple sclerosis (*n* = 112), and mHDGECs (*n* = 48)]. Compared to all other groups, mHDGECs reported significantly lower scores on the QoL domains of psychological functioning and social relationships. For the physical functioning and environment domain, mHDGECs did not significantly differ from the other groups [41].

Two studies compared QoL of HDGECs with a sample of controls (i.e., healthy controls (*n* = 95) and persons at-risk (*n* = 65) [40], and gene-negative siblings (*n* = 36) and partners (*n* = 84) [26]). Both studies found that compared with controls, mHDGECs reported significantly lower QoL (*n* = 31 [40], *n* = 117 [26]) (see Table S4 in the Supplementary materials). No significant differences between pHDGECs (*n* = 118, *n* = 37, respectively) and controls with regard to QoL were found in both the studies [26, 40].

### Health-related quality of life

#### In HDGECs

Eight studies evaluated HRQoL in HDGECs only, without including a control group. Six of these studies assessed HRQoL quantitatively [21–23, 29, 31, 35], whereas two studies explored HRQoL qualitatively with focus groups [36, 37]. No qualitative studies with individual interviews were performed.

Moderate levels of HRQoL were reported by pHDGECs (*n* = 54) [21] and a combined sample of pHDGECs and mHDGECs (*n* = 9, *n* = 23, respectively [23]), whereas low to moderate levels were reported among mHDGECs (*n* = 15, *n* = 84, *n* = 55, respectively [21, 31, 35]). Moreover, several studies found that the HRQoL domains of self-care ability, performance of usual activities, and levels of anxiety/depression were reported to be mostly affected among mHDGECs (*n* = 53, *n* = 55, respectively [29, 35]) and a combined sample of pHDGECs and mHDGECs (*n* = 9, *n* = 23, respectively [23]). In addition, the majority of these groups reported to have some problems with the HRQoL domains of mobility and/or anxiety and depression, followed by pain and discomfort, usual activities, and self-care (see Table S4 in the Supplementary materials for percentages) [23, 29, 35]. HRQoL among mHDGECs tended to decline with increasing disease severity or across disease stages [22, 31, 35], with scores stabilizing in stage IV and V [31]. In the latter study, mHDGECs in advanced disease stages (IV and V) reported the lowest HRQoL, whereas mHDGECs in stage III showed the most wide-ranging health profile ranging from reporting no problems to major problems for the majority of HRQoL domains [31].

One study also included a disease-specific HRQoL questionnaire and found that mHDGECs (*n* = 55) reported lowest scores for the motor domain, followed by the psychology domain [35]. Responses on individual items indicated that participants felt helpless (48%), worried about symptoms (38%), felt handicapped (29%), often dropped objects (22%), or had difficulty with tying laces (20%). Highest scores were reported for the socializing domain, as the majority of participants did not feel ignored (66%), isolated (58%), or no longer invited (66%) [35].

The two qualitative studies explored HRQoL with focus groups with members of the broader HD community, including a subgroup of mHDGECs [36, 37]. Both studies used the same dataset of 6 focus groups with 24 mHDGECs in total. In the first study, social health was considered very relevant to HRQoL in mHDGECs, followed by physical health, emotional health, and cognitive health. Comments related to end-of-life issues were less frequent [36]. With regard to social participation and physical health, participants mostly shared experience regarding the impact of HD on interpersonal relationships, leisure activities, medication, mobility/ambulation, and speech/swallowing difficulties. For emotional and cognitive health, the most shared experiences involved difficulties with executive functioning and learning/memory, as well as aspects of positive psychological functioning and feelings of anxiety/fear. In the other study, separate results were reported for mHDGECs in the early and advanced stage of the disease. mHDGECs in the early-stage (*n* = 8) shared experiences regarding the need to recognize, accept and actively adapt to the increasing demands of chorea and HD, such as physical modifications, taking extra time to manage activities, and volunteering. In contrast, advanced-stage mHDGECs (*n* = 16) described a loss in their autonomy and social participation (e.g., driving, employment, becoming more dependent) due to chorea [37].

#### Statistical comparison between HDGEC subgroups

Two studies assessed HRQoL between different subgroups of HDGECs and found that mHDGECs reported significantly poorer HRQoL as compared to pHDGECs on some HRQoL domains (*n* = 15 and *n* = 54, respectively [21], *n* = 23 and *n* = 9, respectively [23]); see Table S4 in the Supplementary materials.

#### Statistical comparison with other neurological diseases and controls

In total, four studies compared the HRQoL of mHDGECs with that of other patient groups or controls [21, 26, 29, 42]. Two studies included a comparison group of people with other neurological conditions (i.e., cerebellar ataxia, Charcot–Marie–Tooth disease, motor neuron disease, multiple system atrophy, postpolio syndrome, progressive supranuclear palsy [29] and PD [42]). In the first study, conclusions were only drawn for the combined sample of neurological conditions, yet the authors stated that the subgroup of mHDGECs (*n* = 53) reported significantly higher levels on the HRQoL domains of anxiety and depression compared to all other groups (*n* = 213) [29]. In the latter study, no significant differences were found between mHDGECs (*n* = 41) and patients with PD (*n* = 118) on overall HRQoL [42].

Two studies compared HRQoL between HDGECs and gene-negative controls (i.e., non-carriers (*n* = 52) [21], and gene-negative siblings (*n* = 36) and partners (*n* = 84) [26]). Both studies found that mHDGECs (*n* = 15 and *n* = 117, respectively) reported significantly lower levels on nearly all HRQoL domains compared to these controls [21, 26], yet for the latter study, this depended on HD stage (see Table S4 in the Supplementary materials). For pHDGECs (*n* = 54 and *n* = 118, respectively), mixed results were reported across studies, with the former study reporting no significant differences with controls [21], whereas the latter study found pHDGECs to report lower HRQoL compared to controls on the HRQoL domains of bodily pain and general health [26]. Moreover, another study compared HRQoL of mHDGECs (*n* = 41) with that of the general population (n = 123) and found mHDGECs to report significant lower overall HRQoL [42].

### Associated factors of (HR)QoL

Many studies have reported factors associated with (HR)QoL in HDGECS [19–26, 30, 31, 34, 39, 41, 43–45]. Findings from studies that included bivariate analyses were categorized per associated factor and are reported first. See Table 2 for a detailed overview of factors associated with (HR)QoL subdomains. Findings from multivariate/multivariable analyses are reported in a separate paragraph below.

**Table 2:**
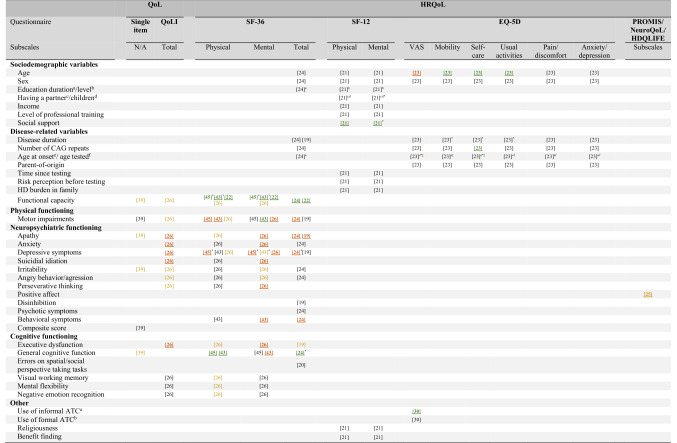
Bivariate findings of factors associated with (HR)QoL in HD

The numbers in the table correspond to the reference numbers in the reference list. For all (HR)QoL measures, a higher score indicates better (HR)QoL, except for the EQ-5D dimensions which display the severity of problems in a domain. For the associated variables, higher scores indicate more of that domain being measured (e.g., more motor impairments, more apathy, better functional capacity, and better cognitive functioning). References displayed in black indicate a non-significant relationship. Bold and underlined references indicate a significant association, with green referring to a positive relationship, whereas red to a negative relationship. Yellow indicates mixed findings, which are described in more detail in the manuscript

*QoL* quality of life, *HRQoL* health-related quality of life, *SF-36* Medical Outcomes Study 36-Item Short Form Health Survey, *SF-12* Medical Outcomes Study 12-Item Short Form Health Survey, *EQ-5D* EuroQol 5D, *PROMIS* Patient-Reported Outcomes Measurement Information System, *HDQLIFE* Huntington disease quality of life, *QoLI* Quality of Life Inventory, *N/A* not applicable, *CAG* cytosine–adenine–guanine, *HD* Huntington’s disease, *ATC* Assistive Technology for Cognition

*Associated factor was uniquely associated with (HR)QoL in multivariable/multivariate analyses. Other uniquely associated variables that were not reported in previous bivariate analyses (and, therefore, not reported in this table) were economic pressure [34], number of CAG repeat, age [43], functional capacity [30, 31], apathy [44], physical symptoms, psychological symptoms, depression-dejection, tension-anxiety [41], level of unmet needs [31]

^a^Informal ATC involves the use of external aids on one's own (e.g., using a cellphone, calendar, and planners)

^b^Formal ATC involves devices and/or software that is designed specifically to support people with cognitive impairment

#### Bivariate findings

1. Sociodemographic characteristics

Higher perceived social support was significantly related to higher mental and physical HRQoL domain scores (henceforth mental and physical HRQoL) in pHDGECs in one study [21]. The same study also found having children to be significantly associated with better mental HRQoL, but not with physical HRQoL in pHDGECs [21]. Mixed results across studies were reported for age. Two studies reported no significant association with age in pHDGECs [21] and mHDGECs [24], whereas one study found lower age to be significantly related to higher HRQoL in a combined group of pHDGECs and mHDGECs with regard to overall HRQoL and the domains of mobility, self-care, and usual activities [23]. No significant associations were found across studies for HRQoL with sex [21, 23, 24], duration or level of education [21, 24], having a partner, income, or level of professional training [21] in pHDGECs [21], mHDGECs [24], or in both groups [23].

None of the included studies investigated associations between QoL and sociodemographic variables.

2. Disease-related characteristics

Higher HRQoL was significantly associated with higher functional capacity among mHDGECs in several studies [22, 24, 26, 43, 45]. Moreover, a lower number of CAG repeats was significantly related to higher HRQoL on the self-care domain among a combined group of pHDGECs and mHDGECs [23]. The same study also found lower age of genetic testing to be significantly associated with higher overall HRQoL and the HRQoL domains of mobility and self-care. No significant associations were found between HRQoL and age of HD onset, parent of origin and disease duration in this study [23]. For mHDGECs, no significant associations were found for HRQoL with disease duration [19, 24], number of CAG repeats, and age of HD onset [24]. For pHDGECs, no significant associations were found for HRQoL with functional capacity [26], time elapsed since testing, risk perception before testing, and HD burden in the family [21].

With regard to QoL, higher functional capacity was related to higher QoL in mHDGECs, but not in pHDGECs [26]. One longitudinal study found higher QoL to be related to higher functional capacity in mHDGECs at baseline, but not at 6-month follow-up or when participants had to recall their own QoL at baseline [39].

3. Physical functioning

Mixed results across studies were reported for motor functioning in mHDGECs, with two studies reporting lower motor impairments to be significantly related to higher HRQoL [24, 26], whereas another study reported no significant relationship [19]. In two studies, the findings varied across HRQoL domains. For physical HRQoL, both of the studies found higher HRQoL to be related to less motor impairments [43, 45]. For mental HRQoL, one study did not find a significant association [45], whereas the other study found a significant positive relationship, indicating that higher mental HRQoL was related to more motor impairments [43]. For pHDGECs, lower motor impairments were associated with higher mental HRQoL, not with physical HRQoL [26].

For QoL, mixed results were reported across studies for motor impairments in mHDGECs, with one study reporting higher QoL to be related to fewer motor impairments [26], whereas another study did not find significant associations [39]. For pHDGECs, no significant associations between QoL and motor impairments were reported [26].

4. Neuropsychiatric functioning

Several neuropsychiatric factors have been investigated as associated factors for HRQoL. For mHDGECs, consistent findings were found for apathy as an associated factor, such that better HRQoL was significantly related to lower levels of apathy [19, 24, 26]. Moreover, fewer behavioral symptoms [24, 43], lower suicidal ideation and less perseverative behavior [26] were significantly related to higher overall [24, 43] and mental HRQoL [24, 26, 43], but not to physical HRQoL in mHDGECs [43]. For depressive symptoms, mixed results have been reported for mHDGECs with three studies reporting fewer depressive symptoms to be related to higher HRQoL [24, 26, 45], whereas one study did not find an association [19]. Another study reported mixed findings depending on the type of depression questionnaire used (i.e., Beck Depression Inventory vs. Hamilton Rating Scale) [43]. Mixed findings were also reported for anxiety, with one study reporting lower levels of anxiety to be associated with higher mental, but not physical HRQoL in mHDGECs [26], whereas another study found no significant associations [24]. No significant associations were found for HRQoL with irritability and angry/aggressive behavior [24, 26], psychotic symptoms [24], and disinhibition [19] in mHDGECs.

For pHDGECs, lower levels of apathy and anxiety, fewer depressive symptoms, lower levels of suicidal ideation and perseverative behavior, and lower levels of irritability and angry/aggressive behavior were significantly related to better mental HRQoL, but not to physical HRQoL [26]. Higher positive affect and well-being was significantly associated with better HRQoL in a combined group of both pHDGECs and mHDGECs, yet moderated by functional capacity such that this association was stronger for HDGECs with higher functional capacity [25].

With regard to QoL, one study showed that higher QoL was related to lower levels of apathy, depressive symptoms, suicidal ideation, and anxiety in both pHDGECs and mHDGECs [26]. In pHDGECs lower levels of irritability, perseverative thinking/behavior, and angry or aggressive behavior were related to higher QoL, whereas this was not the case for mHDGECs [26]. Another study that longitudinally investigated QoL found no significant associations between QoL and overall neuropsychiatric functioning in mHDGECs at any timepoint [39]. Additional post hoc analyses were performed to examine whether specific neuropsychiatric symptoms were related to QoL. At 6-month follow-up, higher QoL was significantly associated with lower irritability frequency, whereas higher QoL at recall was significantly related to lower apathy frequency and severity [39].

5. Cognitive functioning

The majority of studies examining cognitive functioning as a potential associated factor for HRQoL looked at general cognitive functioning and reported mixed results. One study found better cognitive functioning to be significantly related to higher overall HRQoL in mHDGECs [24], whereas two studies reported different findings per HRQoL domain. For physical HRQoL, both studies found higher HRQoL to be significantly associated with better cognitive functioning [43, 45]. For mental HRQoL, one study did not find a significant association [45] whereas the other study found a negative relationship, indicating that higher HRQoL was significantly related to lower cognitive functioning [43].

The relationship of HRQoL with executive functioning was assessed in two studies and findings varied depending on HD subgroup and type of executive measure used. Lower executive dysfunction in everyday behavior, as measured on a self-report questionnaire, was significantly associated with higher HRQoL in mHDGECs [19, 26] and with higher mental, but not physical HRQoL in pHDGECs [26]. Scores on executive tasks were not significantly associated with HRQoL in mHDGECs [19]. In addition, mixed results were reported for scores on different cognitive measures across HD groups. For mHDGECs, mental flexibility and negative emotion recognition were significantly related to higher physical but not mental HRQoL [26], whereas for pHDGECs, these were not significantly associated with HRQoL [26]. Moreover, errors on spatial and social perspective taking tasks and visual working memory were not significantly related with HRQoL in mHDGECs [20, 26]. Visual working memory was, however, significantly related to physical HRQoL in pHDGECs, but not to mental HRQoL [26].

With regard to QoL, one study found higher QoL to be related to lower executive dysfunction in both pHDGECs and mHDGECs, whereas no significant associations were found with visual working memory, mental flexibility and negative emotion recognition [26]. Moreover, one longitudinal study investigated the relationship between QoL and general cognition and found higher cognitive functioning to be significantly related to higher QoL in mHDGECs at baseline, but not at 6-month follow-up or when participants had to recall their own QoL at baseline [39].

6. Other

Several studies also investigated other factors but did not find significant associations of HRQoL with religiousness and benefit finding after genetically confirmed HD in pHDGECs [21], nor with the use of formal assistive technology (i.e., aids/software specifically designed to support individuals with cognitive impairment) in mHDGECs [30]. However, the use of *in*formal assistive technology (i.e., independent use of external aids to support cognitive impairment such as cell phones and calendars), was significantly associated with HRQoL in mHDGECs [30].

#### Multivariate/multivariable findings

Several studies included regression analyses to explore unique determinants of (subdomains of) (HR)QoL [21, 23, 24, 30, 31, 34, 41, 43–45]. For mHDGECs, higher (HR)QoL was uniquely associated with fewer depressive symptoms [24, 41, 43, 45], lower physical symptoms, lower psychological symptoms, lower anxiety levels [41], lower age at HD onset [23], lower economic pressure [34], and a lower level of unmet needs for healthcare and social services [31]. The majority of studies also found higher functional capacity to be uniquely associated with higher HRQoL in mHDGECs [30, 31, 43, 45], whereas only one study examining this factor did not [24]. Mixed results were reported across studies for age [24, 43], cognitive functioning [24, 41, 43], and CAG repeats [23, 43]. Only one study reported a unique association with disease duration in mHDGECs [23], whereas the majority of the included studies examining this factor did not [30, 31, 34, 41, 43].

Consistent findings across studies indicated no unique association of (HR)QoL with the following variables in mHDGECs: motor impairment [24, 45], behavioral problems [24, 43], apathy [24], fatigue-inertia, confusion-bewilderment, control over own body [41], sex [43], income, illness-related expenses, cut backs in spending [34], medication [45], comorbidity [31], informal and formal assistive technology for cognition [30], education, and having an informant during the study [30, 31].

For pHDGECs, higher (HR)QoL was uniquely associated to having children and higher perceived social support, but not to age [21]. In a combined group of both pHDGECs and mHDGECs, a lower level of apathy was uniquely associated to better HRQoL [44].

### Intervention effects on (HR)QoL

Five intervention studies were performed that included (HR)QoL as a primary or main outcome measure [18, 27, 28, 46, 47]. Three out of five studies reported a beneficial intervention effect on (HR)QoL [27, 46, 47].

One study investigated the effects of a 1-year multidisciplinary rehabilitation program in a single group study and found that mHDGECs who completed the program reported significant improvements in their physical HRQoL, but not in their mental HRQoL [27]. The program consisted of 3 in-patient stays of 3 weeks with 8 h of activities held 5 days a week, including daily training exercises with health care providers, group training, patient education sessions, group discussions, and social activities. 31 out of 37 patients (83.8%) completed the full 1-year program. Slight adjustments to medication were made during the intervention period, if necessary, which may have contributed to the observed intervention effects according to the authors. In a small-scale follow-up study, the authors assessed the effects over 2 years among 10 participants who agreed to continue with the same program for an additional year. Six out of 10 participants completed the full 2-year program. Non-significant improvements were found for physical and mental HRQoL on group level [28]. Individual case analysis revealed that five out of six patients reported improved or stable mental and physical HRQoL between baseline and final assessment after 2 years.

Another study investigated the effects of a 4-month respiratory muscle training on pulmonary and swallowing functioning and found that 9 mHDGECs in the respiratory muscle training group showed moderate positive effects on swallowing-related QoL after 4 months of training [46]. This effect was also positive, but smaller, for the control group (*n* = 9) who received less intensive training. There was a 100% full adherence and no adverse events were reported.

A significant and positive effect on HRQoL was also found for a multimodal rehabilitation program in 20 mHDGECs [47]. The program consisted of 25 days of rehabilitation spread across 8 weeks, and included information and education sessions, counseling, and physical training in a group setting. Individual sessions included guidance on social support, insurance issues, and subscription for equipment. The program was well tolerated, as indicated by a relatively low drop-out (i.e., 9.1%) and high attendance rate (i.e., 89.2%). mHDGECs evaluated the program as relevant, effective and reported a high overall satisfaction with the program.

No effects were found for an 8-week Patient Education Program adapted for HD (PEP-HD) in both pHDGECs (*n* = 19) and mHDGECs (*n* = 40) [18]. Both groups tended to improve on mental HRQoL after completion of the program but slightly worsened on physical HRQoL, however, both changes were non-significant. As significant improvements were found on other variables (e.g., behavioral, anxiety, coping), the authors concluded that the PEP was feasible, especially for people with manifest HD. Both groups evaluated the program as good and useful for daily life, with pHDGECs rating the overall program somewhat better than mHDGECs.

## Discussion

To our knowledge, this is the first systematic review to provide a synthesis of the available research on self-reported perceptions of (HR)QoL in HD gene expansion carriers (HDGECs), as well as factors and intervention effects associated with (HR)QoL. Drawing firm conclusions on the included results is complicated by the large variation in research designs and methodological quality across studies. Findings should, therefore, be interpreted with caution.

Across the literature, consistent findings were found for manifest HDGECs (mHDGECs) experiencing lower QoL and HRQoL compared to premanifest HDGECs (pHDGECs) [21, 23, 40] and controls [21, 26, 40, 42]. This is not surprising given the progressive nature of HD affecting various functions essential for participation in daily life, which can ultimately impact (HR)QoL. HRQoL was indeed shown to decline over the course of disease and with increasing disease severity [22, 31, 35]. In line with this, the majority of studies demonstrated that pHDGECs experience a comparable (HR)QoL to that of controls [21, 26, 40]. These findings suggest that the impact of genetic testing and/or the subtle symptoms that might be experienced by pHDGECs, do not greatly affect their (HR)QoL. This might indicate that after genetic testing, long-term psychological adjustment can be present up until the onset of clinical motor symptoms in manifest HD [21]. This is supported by qualitative findings showing that pHDGECs were more negative about the past (and future) as opposed to being positive about the present to which they also related their QoL [32].

Regarding the comparison of (HR)QoL between HDGECs and people with other neurological diseases, findings differed for QoL and HRQoL. Only one study focused on QoL and found that mHDGECs reported worse in terms of psychological and social functioning compared to other neurological diseases [41]. For HRQoL, mixed findings were reported with one study demonstrating mHDGECs to report lower on one HRQoL subdomain (i.e., anxiety and depression) compared to people with other rare long-term neurological conditions [29], whereas another study did not find any differences compared to patients with PD [42]. Given the strong focus on health aspects in HRQoL questionnaires, mHDGECs might not experience more difficulties with regard to these aspects compared to people with other neurological diseases due to the progressive nature and similarities in experienced symptomatology of most of these diseases [48]. The majority of studies indeed showed that mHDGECs evaluated their overall HRQoL as moderate [23, 31, 35]. Problematic areas included mainly the ability for self-care, performance of usual activities, and levels of anxiety/depression [23, 29, 35], common factors also known to be affected in other neurological diseases [48]. When it comes to non-health-related factors (e.g., spiritual, economic, and social) as is the case with QoL, HDGECs might experience more impact as compared to other neurological diseases due to the strong genetic and progressive nature of HD [41] as well as its consequences in terms of social and familial functioning [49, 50].

Moreover, several factors were related to (HR)QoL in HD as summarized in the bivariate findings section above. When looking at the multivariate findings in which other variables were taken into account, some factors were uniquely associated with (HR)QoL and might contribute to the lower experienced (HR)QoL in mHDGECs compared to pHDGECs and controls, including more depressive symptoms [24, 41, 43, 45], worse functional capacity [30, 31, 43, 45], and more physical and psychological symptoms [41], as these factors are known to be inherent to the progressive nature of HD [3, 6]. Other important factors that were uniquely associated with lower (HR)QoL included higher age at HD onset [23], higher economic pressure [34], not having children, lower perceived social support [21], and higher level of unmet needs for healthcare and social services [31]. Mixed findings were reported for apathy [24, 44], cognitive functioning [24, 41, 43], number of CAG repeats [23, 43], disease duration [23, 30, 31, 34, 41, 43], and age [21, 24, 43]. Although some of these factors are static and, therefore, not amenable, the majority of the uniquely associated factors are dynamic and could, therefore, be the subject of intervention/training in order to target (HR)QoL maintenance or improvement (e.g., depressive symptoms, physical and psychological symptoms, perceived social support, and unmet needs). However, only five intervention studies have been conducted with (HR)QoL as a primary outcome measure. Three of these studies reported beneficial effects for (HR)QoL of a multidisciplinary rehabilitation program [27, 47] and a respiratory muscle training [46] in mHDGECs. The multidisciplinary rehabilitation programs that were carried out in two of these studies targeted some of the dynamic factors mentioned above, including physical and cognitive functioning [27, 47], psychological functioning [47], and guidance on social support [47].

The interpretability of the studies included in this review was compromised by several methodological differences and limitations across studies. First, the majority of the studies included a quantitative design, whereas only five studies used a qualitative approach. Although quantitative findings have its advantages (e.g., simplify communication, facilitate generalizability and comparison), qualitative research may provide a deeper understanding of certain constructs and can, therefore, add an important dimension to (HR)QoL research [51]. This is illustrated in the findings of the included qualitative papers, highlighting frequently discussed themes that can be considered important in relation to (HR)QoL (e.g., interpersonal relationships [32, 36, 38], coping with HD status [32], physical health [36], and perseveration of identity, autonomy and personal values [38]). Second, the differences in the (HR)QoL methods used across studies has implications for comparing and interpreting the results. (HR)QoL assessment methods varied from individual interviews and focus groups to single items and full-scale questionnaires. Moreover, the majority of studies included different generic questionnaires to assess (HR)QoL. These types of questionnaires are often used and validated in different populations and allow for appropriate comparison between different conditions. Only two studies included an HD-specific measure (i.e., H-QoL-I [35] and HDQLIFE [25]), which is surprising given the variety of HD-specific questionnaires that have become available [13]. Given the complex nature of HD, a disease-specific measure is more clinically relevant as it can capture features of (HR)QoL specific to HD, yet global features of (HR)QoL may be overlooked [13]. One study used both a disease-specific (H-QoL-I) and generic (EQ-5D) instrument in HD and found moderate to strong relationships between nearly all dimensions of both instruments [35]. mHDGECs reported limitations to their HRQoL in both instruments, yet the disease-specific instrument captured certain features of HD (especially the psychological dimension) better than the generic measure. Third, demographic and clinical data were not always clearly and/or uniformly described across studies (e.g., lack of information on genetic and/or clinical status).

Moreover, based on the quality scores of both appraisal tools (i.e., MMAT [15] and the refined principles of Gill and Feinstein [16, 17]), we can conclude that most studies have both methodological and conceptual shortcomings. Most frequent methodological limitations were not accounting for confounders in the design and analysis and the lack of a representative study sample. With regard to the conceptual clarity, most frequent shortcomings in all studies included the lack of conceptually defining (HR)QoL and the absence of a distinction between QoL and HRQoL. As there is no uniform definition of QoL and HRQoL to date, clarification and differentiation of the used concepts is desired. Moreover, 30% of the included quantitative studies aimed to measure QoL but in fact used a HRQoL measure. This illustrates the mixed use of both terms and the importance of conceptual clarity. An explicit definition of (HR)QoL provides the basis for selecting and clarifying the (HR)QoL domains and instruments considered. However, in the majority of the included quantitative studies, the authors did not explicitly state the (HR)QoL domains they considered important to measure and no adequate reasons for selecting the (HR)QoL instrument(s) of choice were given. It is important to note that good psychometric properties or the wide-use of an instrument are not sufficient reasons for its use [16, 17]. Reasons should cover the suitability of the instrument for its intended use and the instrument’s components should represent the (HR)QoL domains the authors intend to measure [16, 17].

Some limitations of the review process itself should also be acknowledged. Due to the set language constraints, some relevant articles might have been missed. In addition, studies were categorized with regard to study sample (pHDGECs and/or mHDGECs) and concept measured (QoL and/or HRQoL) based on the available information. As the reported information was not always sufficient, the interpretation of and comparison between studies might have been compromised. Moreover, we did not include an assessment of publication bias. However, we believe that the search and screening process was carried out thoroughly and we included all articles irrespective of methodological quality. Moreover, our search was not restricted on publication dates or on (HR)QoL assessment method used. In order to provide a comprehensive overview, we included both QoL and HRQoL (regardless of definition used) as well as quantitative and qualitative research. Both the screening of eligible articles and quality appraisal were independently performed by two authors and two separate tools for appraising the methodological [15] and conceptual clarity [16, 17] were used.

Given the aforementioned methodological limitations, some common themes should be considered in future research regarding (HR)QoL in HD. First, we recommend future (HR)QoL researchers to use the refined criteria of Gill and Feinstein [16, 17] to strengthen their methods and avoid conceptual shortcomings. An explicit distinction between QoL and HRQoL is especially important to ensure a good understanding of the concepts among readers as both terms differ in their intended use. Medical doctors are often interested in the impact of disease on health-related factors such as ability and functioning, whereas for HDGECs, other factors such as spirituality, finance, and culture might be more relevant. Including a clear definition of (HR)QoL and adequate reasons for the domains and instruments of choice allows for more rigorous (HR)QoL research, better comparison between studies, as well as an increase in the overall conceptual understanding. Second, including more qualitative or mixed-methods designs allows for a deeper understanding of the self-reported (HR)QoL and themes HDGECs consider important. In case of solely quantitative research, incorporating both a disease-specific and generic questionnaire is desired as it captures features specific to HD but at the same time allows for comparison between different conditions [13]. As the majority of literature is focused on mHDGECs, more qualitative research on the (HR)QoL in pHDGECs is especially desired. Persons at risk of or tested negative for HD are also an interesting group of study. Moreover, as there is no uniform definition of QoL to date, it would be interesting to explore how HDGECs would define QoL themselves and whether this differs between pHDGECs and mHDGECs. Fourth, this review was limited to self-reported (HR)QoL, yet the progressive nature of HD can compromise the insights affected individuals have into the symptoms they experience. The inclusion of and comparison with proxy-reports should, therefore, be considered [13]. Lastly, more thorough research is still needed with respect to interventions for (HR)QoL in HD. Besides the potential beneficial effects found for multidisciplinary rehabilitation programs, questions remain with regard to placebo-effects and long-term feasibility. Given the many factors that are found to be associated with (HR)QoL, still many other intervention options remain to be explored.

To conclude, (HR)QoL is experienced differently across the course of the disease, with mHDGECs experiencing impaired QoL and HRQoL in comparison to pHDGECs and controls. When compared to other neurological diseases, findings differed for QoL and HRQoL. Although (HR)QoL is key for understanding the consequences of HD and its treatment, there is a tremendous need to improve the methodological and conceptual clarity of (HR)QoL research in HD as medical decision or health care policy makers might rely on these findings.

### Electronic supplementary material

Below is the link to the electronic supplementary material.

Supplementary file1 (DOCX 196 KB)

## Data Availability

No new data were created or analyzed in this study. Data sharing is not applicable.

## Appendix

Collaborating author names HEALTHE-RND consortium: The Netherlands: Niels H. Chavannes, Eline Meijer, Anne-Wil Heemskerk, Erik van Duijn, Susanne T. de Bot, Pearl J. C. van Lonkhuizen; Germany: G. Bernhard Landwehrmeyer, Alzbeta Mühlbäck, Wiebke Frank, Franziska Steck; Czech Republic: Jiří Klempíř, Romama Konvalinková, Eva Bezuchová, Kristýna Dolečková, Olga Klempířová, Jan Roth, Olga Ulmanová; Italy: Ferdinando Squitieri, Sabrina Maffi, Eugenia Scaricamazza, Simone Migliore, Chiara Di Giorgio, Barbara D’Alessio, Melissa Casella; Ireland: Jennifer Hoblyn, Muthukumaran Thangaramanujam, Tom Burke, Emer O’Malley; United Kingdom: Stephen McKenna, Ian McKenna, Jeanette Thorpe, Anna Coffey, Ramona Moldovan, Peter Foley, Jacqueline Kerr.

## Abbreviations

HD: Huntington’s disease
CAG: Cytosine–adenine–guanine
HTT: Huntingtin gene
QoL: Quality of life
HRQoL: Health-related quality of life
WHO: World Health Organization
HDGECs: HD gene expansion carriers
MMAT: Mixed-methods appraisal tool
mHDGECs: Manifest HD gene expansion carriers
pHDGECs: Premanifest HD gene expansion carriers
VAS: Visual analogue scale
PD: Parkinson’s disease
