# Supplementary material for: Quality of life, health-related quality of life, and associated factors in Huntington’s disease: a systematic review
Source: J Neurol. 2023 Jan 30;270(5):2416–37. doi: 10.1007/s00415-022-11551-8 (PMC10129943; doi:10.1007/s00415-022-11551-8)
Supplement: Supplementary file 1 — Supplementary file1 (DOCX 196 KB) [file 415_2022_11551_MOESM1_ESM.docx]

**Quality of life, health-related quality of life, and associated factors in Huntington’s Disease: a systematic review**

Pearl J. C. van Lonkhuizen^1,2,3^, Wiebke Frank^4^, Anne-Wil Heemskerk^1,3^, Erik van Duijn^3,5^, Susanne T. de Bot^6^, Alzbeta Mühlbäck^4,7,8^, G. Bernhard Landwehrmeyer^4^, Niels H. Chavannes^1,2^, and Eline Meijer^1,2^ on behalf of the HEALTHE-RND consortium

^1^ Department of Public Health and Primary Care, Leiden University Medical Center, Leiden, the Netherlands

^2^ National eHealth Living Lab, Leiden University Medical Center, Leiden, the Netherlands

^3^ Huntington Center Topaz Overduin, Katwijk, the Netherlands

^4^ Department of Neurology, University Hospital Ulm, Ulm, Germany

^5^ Department of Psychiatry, Leiden University Medical Center, Leiden, the Netherlands

^6^ Department of Neurology, Leiden University Medical Center, Leiden, the Netherlands

^7^ Department of Neuropsychiatry, kbo-Isar-Amper-Klinikum, Taufkirchen (Vils), Germany

^8^ Department of Neurology and Center of Clinical Neuroscience, 1^st^ Faculty of Medicine, Charles University and General University Hospital, Prague, Czech Republic

Pearl J. C. van Lonkhuizen, MSc

p.j.c.van_lonkhuizen@lumc.nl

Department of Public Health and Primary Care

Leiden University Medical Center

Postzone V0-P, PO Box 9600

2300 RC Leiden, the Netherlands


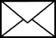


**S1. Search strings**

**Search string PubMed:**

("Huntington Disease"[Mesh] OR "Huntington"[tiab] OR "Huntington's"[tiab] OR "Huntingtons"[tiab]) AND ("Quality of Life"[Mesh] OR "Quality of Life"[tiab] OR "QoL"[tiab] OR "HRQOL"[tiab] OR "life quality"[tiab])

**Search string EMBASE:**

(Huntington chorea/ OR "Huntington".ti,ab. OR "Huntington's".ti,ab. OR "Huntingtons".ti,ab.) AND ("quality of life"/ OR "Quality of Life".ti,ab. OR "QoL".ti,ab. OR "HRQOL".ti,ab. OR "life quality".ti,ab.)

**Search string Web of Science:**

TS=("Huntington" OR "Huntington's" OR "Huntingtons") AND TS=("Quality of Life" OR "QoL" OR "HRQOL" OR "life quality")

**Search string PsycINFO:**

(DE "Huntingtons Disease" OR TX ("Huntington's" OR "Huntingtons")) AND (DE "Quality of Life" OR DE "Health Related Quality of Life" OR DE "Quality of Life Measures" OR TX ("QoL" OR "HRQOL" OR "life quality"))

**Table S2. MMAT quality assessment per study**

| **First author (year)** | **Qualitative studies** | | | | | **Randomized controlled trials** | | | | | **Quantitative non-randomized studies** | | | | | **Quantiative descriptive studies** | | | | | **Quality score** |
| --- | --- | --- | --- | --- | --- | --- | --- | --- | --- | --- | --- | --- | --- | --- | --- | --- | --- | --- | --- | --- | --- |
|  | **1.1** | **1.2** | **1.3** | **1.4** | **1.5** | **2.1** | **2.2** | **2.3** | **2.4** | **2.5** | **3.1** | **3.2** | **3.3** | **3.4** | **3.5** | **4.1** | **4.2** | **4.3** | **4.4** | **4.5** |  |
| Chapman (2002) [33] | + | + | + | - | - |  |  |  |  |  |  |  |  |  |  |  |  |  |  |  | 60% |
| Ready (2011) [32] | + | + | + | + | - |  |  |  |  |  |  |  |  |  |  |  |  |  |  |  | 80% |
| Calvert (2013) [29] |  |  |  |  |  |  |  |  |  |  | - | + | + | - | - |  |  |  |  |  | 40% |
| Carlozzi (2013) [36] | + | + | + | + | + |  |  |  |  |  |  |  |  |  |  |  |  |  |  |  | 100% |
| Chisholm (2013) [40] |  |  |  |  |  |  |  |  |  |  | + | + | - | - | - |  |  |  |  |  | 40% |
| Read (2013) [26] |  |  |  |  |  |  |  |  |  |  | + | + | + | - | + |  |  |  |  |  | 80% |
| Dorey (2016) [35] |  |  |  |  |  |  |  |  |  |  |  |  |  |  |  | + | - | + | - | - | 40% |
| Varda (2016) [23] |  |  |  |  |  |  |  |  |  |  | + | + | + | + | + |  |  |  |  |  | 100% |
| Sherman (2019) [37] | + | + | + | - | - |  |  |  |  |  |  |  |  |  |  |  |  |  |  |  | 60% |
| Exuzides (2022) [42] |  |  |  |  |  |  |  |  |  |  | + | + | + | + | + |  |  |  |  |  | 100% |
| Engels (2022) [38] | + | + | + | + | + |  |  |  |  |  |  |  |  |  |  |  |  |  |  |  | 100% |
| Ready (2008) [39] |  |  |  |  |  |  |  |  |  |  | - | + | + | - | - |  |  |  |  |  | 40% |
| Ho (2009) [45] |  |  |  |  |  |  |  |  |  |  | - | + | - | + | + |  |  |  |  |  | 60% |
| McCabe (2009a) [34] |  |  |  |  |  |  |  |  |  |  | - | + | - | - | + |  |  |  |  |  | 40% |
| Banaszkiewicz (2012) [24] |  |  |  |  |  |  |  |  |  |  | - | + | + | - | + |  |  |  |  |  | 60% |
| Eddy (2013) [19] |  |  |  |  |  |  |  |  |  |  | - | + | - | - | - |  |  |  |  |  | 20% |
| Eddy (2014) [20] |  |  |  |  |  |  |  |  |  |  | - | + | - | - | + |  |  |  |  |  | 40% |
| Brugger (2015) [43] |  |  |  |  |  |  |  |  |  |  | - | + | - | + | + |  |  |  |  |  | 60% |
| van Walsem (2016) [30] |  |  |  |  |  |  |  |  |  |  | - | + | + | + | + |  |  |  |  |  | 80% |
| Fritz (2018) [44] |  |  |  |  |  |  |  |  |  |  | - | + | + | - | + |  |  |  |  |  | 60% |
| Zielonka (2018) [22] |  |  |  |  |  |  |  |  |  |  | + | + | + | + | + |  |  |  |  |  | 100% |
| Ready (2019) [25] |  |  |  |  |  |  |  |  |  |  | + | + | - | + | + |  |  |  |  |  | 80% |
| Licklederer (2008) [21] |  |  |  |  |  |  |  |  |  |  | - | + | + | + | + |  |  |  |  |  | 80% |
| McCabe (2009b) [41] |  |  |  |  |  |  |  |  |  |  | - | + | + | - | + |  |  |  |  |  | 60% |
| van Walsem (2017) [31] |  |  |  |  |  |  |  |  |  |  | - | + | + | + | + |  |  |  |  |  | 80% |
| A'Campo (2012) [18] |  |  |  |  |  |  |  |  |  |  | + | + | - | - | + |  |  |  |  |  | 60% |
| Piira (2013) [27] |  |  |  |  |  |  |  |  |  |  | + | - | + | - | + |  |  |  |  |  | 60% |
| Piira (2014) [28] |  |  |  |  |  |  |  |  |  |  | + | - | - | - | + |  |  |  |  |  | 40% |
| Reyes (2015) [46] |  |  |  |  |  | + | + | + | - | + |  |  |  |  |  |  |  |  |  |  | 80% |
| Ringqvist (2021) [47] |  |  |  |  |  |  |  |  |  |  | + | + | + | + | + |  |  |  |  |  | 100% |

Note. MMAT criteria: 1.1: Qualitative approach is appropriate to address the aim; 1.2: Qualitative data collection methods is adequate to address the aim; 1.3: Findings are adequately derived from the data; 1.4: Interpretation of results is sufficiently substantiated by the data; 1.5: There is coherence between the data sources, collection, analyses and interpretation; 2.1: Randomization is appropriately performed; 2.2: Groups are comparable at baseline; 2.3: There is complete outcome data; 2.4: The outcome assessors are blinded to the intervention provided; 2.5: Participants adhered to the assigned intervention; 3.1: Participants are representative of the target population; 3.2: Measurements used are appropriate with regard to both the outcome and intervention (or exposure); 3.3: There is complete outcome data; 3.4: There is accounted for confounders in the design and analyses; 3.5: Statistical analysis is appropriate to address the aim (note that the authors substituted the original item 3.5 ‘Intervention is administered (or exposure occurred) as intended’ with the current criterion as this was considered more appropriate for the quantitative non-randomized and non-intervention studies included in this review); 4.1: Sampling strategy is relevant to address the aim; 4.2: Sample is representative of the target population; 4.3: Measurements used are appropriate; 4.4: Risk of non-response bias is low; 4.5: Statistical analysis is appropriate to address the aim.

+ Article met the criterion.

- Article did not meet the criterion. In case no appropriate or clear information was reported to appraise the criterion (e.g., ‘can’t tell’), the article was rated as not meeting that criterion.

**Table S3. Conceptual and methodological clarity of (HR)QoL studies**

| **First author (year)** | **Conceptual definition (HR)QoL** | **Defined domains (HR)QoL** | **Reason choice instrument** | **Aggregation into composite score** | **Patient rating over-all (HR)QoL** | **Distinction between QoL and HRQoL** | **Possibility of supplementing items** | **If so, supplemental items incorporated** | **Possibility of rating importance items** | **If so, importance ratings incorporated** | **Quality score** (%) |
| --- | --- | --- | --- | --- | --- | --- | --- | --- | --- | --- | --- |
| Chapman (2002) [33] | - | NA | NA | NA | NA | - | NA | NA | NA | NA | 0% |
| Ready (2011) [32] | - | NA | NA | NA | NA | - | NA | NA | NA | NA | 0% |
| Calvert (2013) [29] | - | - | - | - | + | - | - | NA | - | NA | 13% |
| Carlozzi (2013) [36] | + | NA | NA | NA | NA | + | NA | NA | NA | NA | 100% |
| Chisholm (2013) [40] | - | - | - | - | + | - | - | NA | - | NA | 13% |
| Read (2013) [26] | + | - | - | + | - | - | - | NA | - | NA | 25% |
| Dorey (2016) [35] | - | - | - | + | - | - | - | NA | - | NA | 13% |
| Varda (2016) [23] | - | - | + | + | + | - | - | NA | - | NA | 38% |
| Sherman (2019) [37] | - | NA | NA | NA | NA | - | NA | NA | NA | NA | 0% |
| Exuzides (2022) [42] | - | - | - | + | + | - | - | NA | - | NA | 25% |
| Engels (2022) [38] | + | NA | NA | NA | NA | - | NA | NA | NA | NA | 50% |
| Ready (2008) [39] | + | - | + | - | + | + | - | NA | - | NA | 50% |
| Ho (2009) [45] | + | - | - | + | - | - | - | NA | - | NA | 25% |
| McCabe (2009a) [34] | - | - | + | + | -* | - | - | NA | - | NA | 25% |
| Banaszkiewicz (2012) [24] | - | - | - | + | - | - | - | NA | - | NA | 13% |
| Eddy (2013) [19] | - | - | + | + | - | - | - | NA | - | NA | 25% |
| Eddy (2014) [20] | - | - | - | + | - | - | - | NA | - | NA | 13% |
| Brugger (2015) [43] | - | + | - | + | - | - | - | NA | - | NA | 25% |
| van Walsem (2016) [30] | - | - | - | - | + | - | - | NA | - | NA | 13% |
| Fritz (2018) [44] | - | - | - | + | - | - | - | NA | - | NA | 13% |
| Zielonka (2018) [22] | - | - | - | + | - | - | - | NA | - | NA | 13% |
| Ready (2019) [25] | - | - | - | + | - | - | - | NA | - | NA | 13% |
| Licklederer (2008) [21] | - | + | - | + | - | - | - | NA | - | NA | 25% |
| McCabe (2009b) [41] | - | - | - | + | -* | - | - | NA | - | NA | 13% |
| van Walsem (2017) [31] | + | - | - | + | + | - | - | NA | - | NA | 38% |
| A'Campo (2012) [18] | - | + | - | + | - | - | - | NA | - | NA | 25% |
| Piira (2013) [27] | - | - | - | + | - | - | - | NA | - | NA | 13% |
| Piira (2014) [28] | - | - | - | + | - | - | - | NA | - | NA | 13% |
| Reyes (2015) [46] | - | - | - | + | - | - | - | NA | - | NA | 13% |
| Ringqvist (2021) [47] | - | - | - | - | + | - | - | NA | - | NA | 13% |

Abbreviations. QoL: Quality of Life; HRQoL: Health-related Quality of Life; NA: Not applicable.

+ Article met the criterion.

- Article did not meet the criterion.

* This option is part of the official measure, but is not included in the study.

**Table S4. Reported mean scores and standard deviations on (HR)QoL questionnaires per study**

| **First author**  **(year)** | **Stratifying variable** | **Questionnaire**  (subscales) | | | | | | | | | | |  |  |  |  |  |
| --- | --- | --- | --- | --- | --- | --- | --- | --- | --- | --- | --- | --- | --- | --- | --- | --- | --- |
|  |  | **SF-36/SF-12** (higher score indicates better HRQoL) | | | | | | | | | | |  |  |  |  |  |
|  |  | PhyF | RF(p) | BP | GH | VIT | SF | RF(e) | MH | PSs | MSs | Total |  |  |  |  |  |
| Licklederer  (2008) [21] | pHDGECs | - | - | - | - | - | - | - | - | 54(6)^∇^ | 46(12)^∇^ | - |  |  |  |  |  |
|  | mHDGECs | - | - | - | - | - | - | - | - | 46(8)*^∇^ | 40(12)*^∇^ | - |  |  |  |  |  |
| Ho  (2009) [45] | mHDGECs | 67(34) | 51(45) | 85(23) | 57(24) | 59(24) | 62(22) | 58(48) | 72(20) | 26(39) | 19 (42) | - |  |  |  |  |  |
| A'Campo  (2012) [18] | pHDGECs | - | - | - | - | - | - | - | - | 52(9)^∇^ | 44(9) | - |  |  |  |  |  |
|  | mHDGECs | - | - | - | - | - | - | - | - | 46(10) | 40(12) | - |  |  |  |  |  |
| Eddy  (2013) [19] | mHDGECs | - | - | - | - | - | - | - | - | - | - | 92(21) |  |  |  |  |  |
| Eddy  (2014) [20] | mHDGECs | - | - | - | - | - | - | - | - | - | - | 93(26) |  |  |  |  |  |
| Piira  (2013) [27] | Baseline | - | - | - | - | - | - | - | - | 44(8) | 52(11) | - |  |  |  |  |  |
|  | 1-year FU | - | - | - | - | - | - | - | - | 49(8)** | 53(11) | - |  |  |  |  |  |
| Piira  (2014) [28] | Baseline | - | - | - | - | - | - | - | - | 73(NR) | 81(NR) | - |  |  |  |  |  |
|  | 15-month FU | - | - | - | - | - | - | - | - | 85(NR) | 92(NR) | - |  |  |  |  |  |
|  | 2-year FU | - | - | - | - | - | - | - | - | 86(NR) | 91(NR) | - |  |  |  |  |  |
| Read  (2013) [26] | pHDGECs  (group A^a^) | 94(13) | 86(21) | 86(19)* | 70(18)* | 66(19) | 84(21) | 86(21) | 73(19) | 55(8) | 48(11) | - |  |  |  |  |  |
|  | pHDGECs (group B^b^) | 94(16) | 87(19) | 89(21) | 68(19)* | 63(19) | 83(23) | 85(19) | 72(16) | 55(7) | 47(10) | - |  |  |  |  |  |
|  | mHDGECs (group C^c^) | 91(14) | 82(22)* | 86(21)* | 66(21)* | 67(19) | 80(22)* | 81(22) | 73(15) | 54(7) | 47(10) | - |  |  |  |  |  |
|  | mHDGECs (group D^d^) | 68(28)* | 52(30)* | 78(28) | 59(18)* | 55(23)* | 57(28)* | 54(31)* | 67(22)* | 47(11)* | 40(13)* | - |  |  |  |  |  |
| Brugger  (2015) [43] | mHDGECs | 78(30) | 75(37) | 83(17) | 54(19) | 57(14) | 48(11) | 83(34) | 61(11) | 38(7) | 46(7) | - |  |  |  |  |  |
| Zielonka  (2018) [22] | Female | - | - | - | - | - | - | - | - | - | - | 99(24) |  |  |  |  |  |
|  | Male | - | - | - | - | - | - | - | - | - | - | 103(22) |  |  |  |  |  |
|  |  | **WHOQOL-BREF** (higher score indicates better QoL) | | | | | | | | | | |  |  |  |  |  |
|  |  | PhyH | PsyH | SR | EN | Total |  |  |  |  |  |  |  |  |  |  |  |
| McCabe (2009a) [34] | Baseline | - | - | - | - | 50(20) |  |  |  |  |  |  |  |  |  |  |  |
|  | 12-month FU | - | - | - | - | 49(21) |  |  |  |  |  |  |  |  |  |  |  |
| McCabe (2009b) [41] | mHDGECs | 48(21) | 48(23)* | 49(22)* | 64(19) | 52(17) |  |  |  |  |  |  |  |  |  |  |  |
|  |  | **QoLI** (higher score indicates better QoL) | | | | | | | | | | |  |  |  |  |  |
|  |  | HF | SE | P/S | FL | Total |  |  |  |  |  |  |  |  |  |  |  |
| Read  (2013) [26] | pHDGECs  (group A^a^) | 23(5) | 25(4) | 22(6) | 23(6) | 23(5) |  |  |  |  |  |  |  |  |  |  |  |
|  | pHDGECs (group B^b^) | 22(5) | 24(5) | 22(5) | 23(6) | 23(5) |  |  |  |  |  |  |  |  |  |  |  |
|  | mHDGECs (group A^c^) | 22(5) | 24(4) | 23(4) | 23(7)* | 23(4) |  |  |  |  |  |  |  |  |  |  |  |
|  | mHDGECs (group B^d^) | 19(6)* | 22(4) | 21(6) | 23(6) | 21(5)* |  |  |  |  |  |  |  |  |  |  |  |
|  |  | **H-QoL-I** (higher score indicates better HRQoL) | | | | | | | | | | |  |  |  |  |  |
|  |  | Motor | PSY | SOC | Total |  |  |  |  |  |  |  |  |  |  |  |  |
| Dorey  (2016) [35] | mHDGECs | 57(NR) | 58(NR) | 81(NR) | 66(NR) |  |  |  |  |  |  |  |  |  |  |  |  |
|  |  | **EQ-5D** (percentages display severity of problems in the 5 domains (% none; % some/major problems). For VAS and utility scores, a higher score indicates better health) | | | | | | | | | | | | | | | |
|  |  | MOB | SC | UA | P/D | A/D | VAS | Utility score |  |  |  |  |  |  |  |  |  |
| Calvert  (2013) [29] | mHDGECs | NR;92 | NR21;79 | 8;92 | 38;62 | 13;87 | - | - |  |  |  |  |  |  |  |  |  |
| Dorey  (2016) [35] | mHDGECs | 38;62 | 47;53 | 38;62 | 69;31 | 37;63 | - | 0.5(0.4) |  |  |  |  |  |  |  |  |  |
| van Walsem  (2016) [30] | mHDGECs | - | - | - | - | - | 52(26) | - |  |  |  |  |  |  |  |  |  |
| van Walsem  (2017) [31] | mHDGECs | 35;65 | 44;56 | 25;75 | 49;51 | 32;68 | 52(26) | - |  |  |  |  |  |  |  |  |  |
| Varda  (2016) [23] | pHDGECs | 100;0^∇^ | 100;0^∇^ | 78;22^∇^ | 44;56 | 22;78 | - | - |  |  |  |  |  |  |  |  |  |
|  | mHDGECs | 9;91^∇^ | 22;78^∇^ | 17;83^∇^ | 57;43 | 26;74 | - | - |  |  |  |  |  |  |  |  |  |
| Ringqvist (2021) [47] | Baseline | - | - | - | - | - | 67(21) | - |  |  |  |  |  |  |  |  |  |
|  | 8-week FU | - | - | - | - | - | 77(17)** | - |  |  |  |  |  |  |  |  |  |
| Exuzides (2022) [42] | mHDGECs | - | - | - | - | - | 59(23)* | 0.7(0.2)* |  |  |  |  |  |  |  |  |  |
|  |  | **PROMIS (3), Neuro-QoL (8), HDQLIFE (5)** (higher score indicates more of the domain being measured) | | | | | | | | | | | | | | | |
|  |  | D | A | Anger | PAW | SSRA | PSRA | EF | Stigma | UEF | LEF | EBD | Chorea | SpD | SwD | CDD | MP |
| Ready  (2019) [25] | pHDGECs | 49(10) | 53(10) | 48(12) | 55(9) | 51(8) | 50(8) | 44(10) | 46(8) | 50(8) | 54(8) | 46(11) | 43(7) | 45(7) | 46(7) | 50(9) | 49(10) |
|  | early mHDGECs | 51(11) | 53(10) | 48(12) | 55(9) | 48(8) | 47(8) | 38(9) | 52(8) | 43(9) | 47(9) | 47(10) | 53(7) | 51(8) | 52(8) | 51(10) | 50(10) |
|  | late mHDGECs | 51(11) | 54(11) | 47(13) | 54(9) | 44(8) | 43(8) | 29(8) | 53(10) | 32(7) | 38(8) | 47(12) | 57(7) | 55(8) | 56(8) | 50(11) | 49(8) |
|  |  | **SWAL-QoL** (higher score indicates better QoL) | | | | | | | | | | | | | | | |
|  |  | Total |  |  |  |  |  |  |  |  |  |  |  |  |  |  |  |
| Reyes  (2015) [46] | IG  baseline to 4-month FU | 83(8)  to 89(13) |  |  |  |  |  |  |  |  |  |  |  |  |  |  |  |
|  | CG  baseline to 4-month FU | 71(13) to  77(15) |  |  |  |  |  |  |  |  |  |  |  |  |  |  |  |
|  |  | **QoL single item** (ranging from 1: bad QoL to 5: excellent QoL) | | | | | | | | | | | | | | | |
|  |  | NA |  |  |  |  |  |  |  |  |  |  |  |  |  |  |  |
| Ready  (2008) [39] | Baseline | 3(1) |  |  |  |  |  |  |  |  |  |  |  |  |  |  |  |
|  | 6-month FU | 3(1) |  |  |  |  |  |  |  |  |  |  |  |  |  |  |  |
|  | Recall of baseline at 6-month FU | 3(1)** |  |  |  |  |  |  |  |  |  |  |  |  |  |  |  |
| Chisholm  (2013) [40] | pHDGECs | 4(1) |  |  |  |  |  |  |  |  |  |  |  |  |  |  |  |
|  | mHDGECs | 4(1)*^∇^ |  |  |  |  |  |  |  |  |  |  |  |  |  |  |  |

Note. Rounded means and standard deviations are reported, unless otherwise stated (i.e., EQ-5D). The following included articles did not report means or standard deviations on a (HR)QoL questionnaire: [24] and [44], as well as the studies including a qualitative design [32, 33, 36-38].

Abbreviations. SF-36: Medical Outcomes Study 36-Item Short Form Health Survey; SF-12: Medical Outcomes Study 12-Item Short Form Health Survey; HRQoL: Health-Related Quality of Life; PhyF: physical functioning; RF(p): role functioning physical; BP: bodily pain; GH: general health; VIT: vitality; SF: social functioning; RF(e): role functioning emotional; MH: mental health; PSs: physical summary score; MSs: mental summary score; pHDGECs: premanifest Huntington's Disease Gene Expansion Carriers; mHDGECs: manifest Huntington's Disease Gene Expansion Carriers; FU: follow-up; WHOQoL-BREF: World Health Organization Quality of Life Questionnaire short version; QoL: Quality of Life; PhyH: physical health; PsyH: psychological health; SR: social relationships; EN: environment; QoLI: Quality of Life Inventory; HF: health and function; SE: social and economic; P/S: psychological and spiritual; FL: family life; H-QoL-I: Huntington Quality of Life Instrument; PSY: psychology; SOC: socializing; EQ-5D: EuroQol 5D; MOB: mobility; SC: self-care; UA: usual activities; P/D: pain and discomfort; A/D: anxiety and depression; VAS: visual analogue scale; PROMIS; Patient-Reported Outcomes Measurement Information System; HDQLIFE: Huntington Disease Quality of Life; WHODAS: WHO Disability Assessment Schedule; D: depression; A: anxiety; PAW: positive affect and well-being; SSRA: satisfaction with social roles and activities; PSRA: participation in social roles and activities; EF: executive function; UEF: upper extremities function; LEF: lower extremities function; EBD: emotional/behavioral dyscontrol; SpD: speech difficulties; SwD: swallowing difficulties; CDD: concerns with death and dying; MP: meaning and purpose; SWAL-QoL: Swallow Quality of Life Questionnaire; IG: intervention group; CG: control group

^a^ pHDGECs furthest from predicted age of HD onset

^b^ pHDGECs closest to predicted age of HD onset

^c^ mHDGECs in early stage I of the disease

^d^ mHDGECs in early stage II of the disease

* Significant difference with controls/other patient groups (see text).

^∇^ Significant difference between included HD groups. Due to rounding, some mean scores might appear the same, but differ significantly from each other (e.g., [40]).

** Significant difference between time points
